# The role of birth month in the burden of hospitalisations for acute lower respiratory infections due to respiratory syncytial virus in young children in Croatia

**DOI:** 10.1371/journal.pone.0273962

**Published:** 2022-09-02

**Authors:** You Li, Ena Batinović, Petra Milić, Joško Markić

**Affiliations:** 1 Centre for Global Health, Usher Institute, University of Edinburgh, Edinburgh, United Kingdom; 2 School of Public Health, Nanjing Medical University, Nanjing, China; 3 Department of Pediatrics, University Hospital Centre Split, Split, Croatia; 4 School of Medicine, University of Split, Split, Croatia; Public Library of Science, UNITED STATES

## Abstract

**Background:**

Birth month was an important risk factor for respiratory syncytial virus (RSV) hospitalisation in infants. However, little is known about the role of birth month in RSV hospitalisation in finer age bands during infancy, which is relevant to strategies for RSV passive immunisations for infants. We aimed to understand the role of birth month in the burden of RSV-associated acute lower respiratory infection (ALRI) hospitalisation in finer age bands of the first year of life.

**Methods:**

In this retrospective study, we analysed the hospitalisation records during 2014–19 at the University Hospital of Split, Split-Dalmatia County, Croatia. We estimated all-cause and RSV associated ALRI hospitalisation rates among children under five years, with a focus on infants by finer age band and birth month.

**Results:**

We included 1897 ALRI hospitalisations during the study period. Overall in children under five years, annual hospitalisation rate was 14.66/1000 (95% CI: 14.01–15.34) for all-cause ALRI, and was 7.56/1000 (95% CI: 6.83–8.34) for RSV-ALRI. RSV-ALRI hospitalisation rate was highest in infants aged 28 days–<3 months (61.15/1000, 95% CI: 52.91–70.31). Infants born in November, December and January (2–3 months before RSV peak) had the highest hospitalisation rates during infancy. Depending on the birth month of infants, the risk of RSV-ALRI hospitalisation peaked at different months of age; infants who were born in September had the highest RSV-ALRI hospitalisation rate at the age of 3–<6 months.

**Conclusions:**

Our study underlines the importance of birth month in planning RSV immunisation strategies for infants, and provides useful baseline data for effectiveness analysis of novel RSV prophylactic products.

## Introduction

Respiratory syncytial virus (RSV) is an important viral cause of acute lower respiratory infection (ALRI), such as pneumonia and bronchiolitis, in young children [1–3]. Globally, RSV was associated with 33.1 million ALRI episodes, 3.2 hospital admissions and 59600 in-hospital deaths in children under five years in 2015; 44% of those hospitalisations and 46% of the in-hospital deaths occurred in infants younger than six months [4]. RSV has clear seasonal epidemics in most regions and usually peaks in winter months in the temperate regions [5].

Currently, the only licensed RSV prophylaxis is a specific RSV monoclonal antibody, Palivizumab (Synagis), which needs to be administered on a monthly basis for five months during the RSV season. Due to its high costs, Palivizumab is administered mainly to high-risk infants and almost exclusively in high-income countries. Nonetheless, there are many other RSV prophylactic products under development, with several of them at phase 3 clinical trial [6], including a long-acting RSV monoclonal antibody, Nirsevimab (Sanofi/AstraZeneca) and a maternal RSV vaccine, ResVax (Novavax). Both Nirsevimab and ResVax are designed to provide passive immunisations for infants aged six months or younger with protection of 3–5 months [7, 8]; given the limited duration of protection, these RSV prophylactics will need to be timed carefully to the expected RSV risk time window of the infants.

Previous studies from the US [9] and the UK [10] highlighted the important role of birth month in the risk of RSV-hospitalisation during the first year of life. More data are warranted to understand how RSV-hospitalisation rate could vary by birth month in finer age bands (e.g. 0-<28 days, 1-<3 months, etc.), which is important to refine the targeted chronological age as well as time window for administering novel RSV passive immunisation products.

In the present study, we aimed to estimate the burden RSV-ALRI hospitalisation among young children residing in the Split-Dalmatia County, Croatia and to understand the role of birth month in determining RSV-ALRI hospitalisation rate by finer age band among infants.

## Methods

### Study design and setting

We conducted a retrospective population-based study among all children younger than five years of age who were hospitalised for acute lower respiratory infection (ALRI) at the University Hospital of Split during 2014–19. The University Hospital of Split is the only hospital that serves the Split-Dalmatia county. The Split-Dalmatia County is in south of Croatia. In 2019, the total population of Split-Dalmatia county is 447.7 thousand, with 20.6 thousand (4.6%) being under five years of age. The study was approved by the Ethics Committee of the University Hospital of Split. All data were fully anonymised before accessing them for analysis. The Ethics Committee of the University Hospital of Split waived the requirement for informed consent.

### Data collection

We reviewed the hospitalisation records at the University Hospital of Split between 1^st^ Jan 2014 and 31^st^ Dec 2019, and we extracted all hospitalisation records of ALRI (including pneumonia and bronchiolitis / bronchitis) in children under five years according to the discharge summary medical report. We defined severe ALRI as ALRI with hypoxemia (defined as peripheral capillary oxygen saturation, SpO2 < 90%), and defined very severe ALRI as ALRI with one danger sign (cyanosis, difficulty in breastfeeding or drinking, vomiting everything, convulsions, lethargy, or unconsciousness, head nodding), mechanical ventilation, or intensive care unit (ICU) admission [11].

Information on laboratory testing for RSV was also obtained to aid the estimate of RSV-associated ALRI hospitalisations. Testing for RSV was ordered at the discretion of the physicians. Nasal aspirate specimens were collected from the patients immediately upon admission and tested using rapid antigen test (RSV-AdenoGnost RESP, BioGnost, Croatia) on the same day (or the following morning for midnight admission). Over the study period, there was no major change in RSV testing practices except for the year 2015 due to financial/technical reasons at the hospital.

### Data analysis

#### Hospitalisation rates

Annualised hospitalisation rates of all-cause ALRI and RSV-ALRI were estimated for different age groups, including <60 months, <12 months and 12–<60 months as well as five finer age groups for the infant group, 0–27 days, 28 days–<3 months, 3–<6 months, 6–<9 months and 9–<12 months. Confidence intervals were calculated using the exact method by Ulm [12]. For RSV-ALRI hospitalisation rate, we assumed that the RSV positivity rate among ALRI not being tested for RSV was equal to that among ALRI being tested for RSV, for each age group per calendar month. This allowed us to adjust for the absence of RSV testing while accounting for factors including age and calendar month that accounted for the most variations in RSV positivity rate. For the adjustment, we selected to downscale the population denominator using the proportion of RSV testing, which could reflect the increased statistical uncertainty due to the lack of RSV testing, rather than upscaling the numerator (i.e. RSV positive hospitalisations) [13]. The adjustment above was conducted by age group and calendar month. In addition, we estimated the number of RSV-ALRI hospitalisation in children under five years in Croatia for the year 2019, by applying the hospitalisation rate of RSV-ALRI in the Split-Dalmatia county to the whole population of Croatia. The population denominator for the Split-Dalmatia county and for Croatia nationwide was extracted from the national yearbook [14].

#### RSV seasonality

RSV seasonality was defined in two different approaches, based on either RSV positivity rate or the absolute number of RSV counts. For the first approach, we defined high RSV activity months as any months with RSV proportion positive of >10% [15]. This could be used for assessing RSV season for each year or for assessing the average RSV season across the study years. For the second approach, we totalled up the absolute RSV counts by month across the study years and calculated the annual average percentage (AAP) of RSV counts for each month, [5, 16, 17]; we defined peak RSV activity months as the top three months with the highest AAP. This could be used only for assessing the average RSV season.

#### Stratified analysis by birth month

We conducted stratified analysis of hospitalisation rates for all-cause ALRI and RSV-ALRI by birth month, with a focus on the first year of life. For each of the twelve birth months, we used the number of live births as the denominator, which was extracted from hospital records, and calculated the hospitalisation rate per 1000 live births in the first year of life. This was further stratified by finer age group (i.e. 0–27 days, 28 days–<3 months, 3–<6 months, 6–<9 months and 9–<12 months). We reported only the unadjusted hospitalisation rates for all-cause ALRI and RSV-ALRI for the twelve birth months (rather than further adjusting for other potential confounding factors such as birth weight and prematurity, if the prevalence of these factors differed by month) as the unadjusted estimates were deemed more relevant to the implementation of RSV immunisation programmes.

In addition, we conducted correlation analyses to assess the association between the birth month associated with the highest/lowest RSV-ALRI hospitalisation rates in the first year of life (hereafter referred to as “birth month with peak/trough RSV”) and the calendar month with the highest/lowest RSV proportion positive (hereafter referred to as “calendar month with peak/trough RSV”). This was done by a series of Pearson’s correlation analyses between the time series of RSV-ALRI hospitalisation rates by birth month and the time series of RSV proportion positive, using different lags in months. The time lag with the highest positive correlation coefficient was then regarded as the time lag between birth month with peak RSV and calendar month with peak RSV. The time lag with the highest (absolute value) negative correlation coefficient was regarded as the time lag between birth month with trough RSV and calendar month with peak RSV. As a sensitivity analysis, we replaced RSV proportion positive with AAP in the correlation analysis.

#### Statistical software

All data analyses and visualisation were conducted using the R software (version 4.0.3).

## Results

Between 1^st^ Jan 2014 and 31^st^ Dec 2019, we included a total of 1897 ALRI hospitalisations in the analysis, of which 131 (6.9%) and 49 (2.6%) were severe and very severe ALRI, respectively.

The overall proportion of testing for RSV among ALRI hospitalisations was 40.5% (768/1897). Testing for RSV was low (12.0%) in the year 2015 due to financial/technical reasons, and the proportion of testing ranged 32.4%–55.7% among the rest of the study years (**S1 Table**). Over 60% (660/1045, 63.2%) of ALRI cases in infants were tested for RSV; by contrast, only 12.7% (108/852) of ALRI cases in children aged 12–<60 months were tested for RSV (**S1 Table**). In infants, the proportion of testing ranged 42.9%–77.0% among different birth months (**S2 Table**).

### Burden of all-cause ALRI and RSV-ALRI hospitalisations

**Fig 1:**
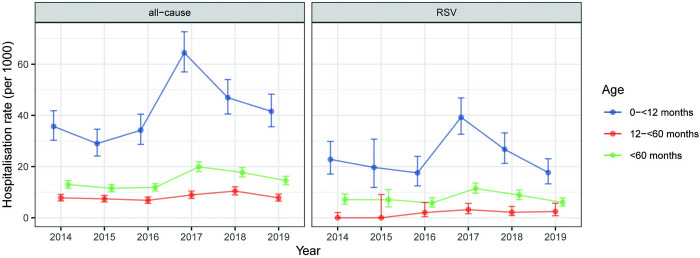
Year-on-year variations on annual hospitalisation rates of all-cause and RSV-associated ALRI in children under five years of age. RSV = respiratory syncytial virus; ALRI = acute lower respiratory infection.

The overall all-cause ALRI hospitalisation rate in children under five years across the study years was 14.66 per thousand per year (95% CI: 14.01–15.34) for ALRI of any severity, 1.01 per thousand per year (0.85–1.20) for severe ALRI, and 0.38 per thousand per year (0.28–0.50) for very severe ALRI. The overall RSV-ALRI hospitalisation rate in children under five years across the study years was 7.56 per thousand per year (95% CI: 6.83–8.34) for ALRI of any severity, 0.39 per thousand per year (0.24–0.60) for severe ALRI, and 0.18 per thousand per year (0.09–0.33) for very severe ALRI. RSV-ALRI hospitalisation rate was higher in infants (<12 months) than children aged 12–<60 months, and was highest in infants aged 28 days–<3 months (61.15 per thousand per year, 95% CI: 52.91–70.31) (**Table 1**). The overall RSV proportion positive was 51.6% (396/768) and peaked at the age of 28 days–<3 months (77.6%, 318/410) (**S3 Table**). RSV-ALRI hospitalisation rate in children under five years was highest in 2017 (11.44 per thousand, 95% CI: 9.59–13.56) and was lowest in 2016 (5.79 per thousand, 95% CI: 4.17–7.83); similar year-on-year variations were found for infants and for all-cause ALRI (**Fig 1**). By applying the RSV-ALRI hospitalisation rate in the Split-Dalmatia county, we estimated that there were 1100 (95% CI: 840–1420) RSV-ALRI hospitalisations among children under five years in Croatia in the year 2019.

**Table 1:** Annual hospitalisation rate (per thousand) of all-cause and RSV-associated ALRI by age group in 2014–19.

Age	ALRI	Severe ALRI	Very severe ALRI
**All-cause**			
<28d	37.07 (29.26–46.34)	4.33 (1.98–8.23)	3.85 (1.66–7.59)
28d–<3m	98.70 (89.38–108.74)	4.57 (2.75–7.14)	2.17 (0.99–4.11)
3–<6m	49.11 (43.76–54.93)	2.73 (1.59–4.37)	1.44 (0.66–2.74)
6–<9m	25.52 (21.71–29.81)	1.77 (0.88–3.16)	0.80 (0.26–1.87)
9–<12m	14.93 (12.05–18.29)	0.96 (0.35–2.10)	0.16 (<0.01–0.89)
**<12m**	41.93 (39.43–44.55)	2.49 (1.91–3.19)	1.28 (0.88–1.81)
12–<60m	8.16 (7.62–8.72)	0.66 (0.51–0.84)	0.16 (0.09–0.26)
**<60m**	14.66 (14.01–15.34)	1.01 (0.85–1.20)	0.38 (0.28–0.50)
**RSV-associated**			
<28d	24.95 (17.38–34.70)	2.17 (0.05–12.07)	1.93 (0.05–10.73)
28d–<3m	61.15 (52.91–70.31)	2.89 (1.49–5.05)	1.44 (0.53–3.14)
3–<6m	25.90 (20.98–31.63)	0.91 (0.19–2.66)	0.96 (0.12–3.48)
6–<9m	10.61 (7.25–14.97)	0.76 (0.16–2.21)	0.40 (0.01–2.24)
9–<12m	4.81 (2.31–8.85)	0.24 (0.01–1.34)	0 (0–0.59)
**<12m**	23.51 (21.17–26.03)	1.21 (0.74–1.87)	0.76 (0.36–1.39)
12–<60m	1.96 (1.28–2.88)	0.05 (<0.01–0.28)	0 (0–0.15)
**<60m**	7.56 (6.83–8.34)	0.39 (0.24–0.60)	0.18 (0.09–0.33)

RSV = respiratory syncytial virus; ALRI = acute lower respiratory infection. Numbers are presented as hospitalisation rate per 1000 children, with 95% confidence intervals.

### RSV seasonality

**Fig 2:**
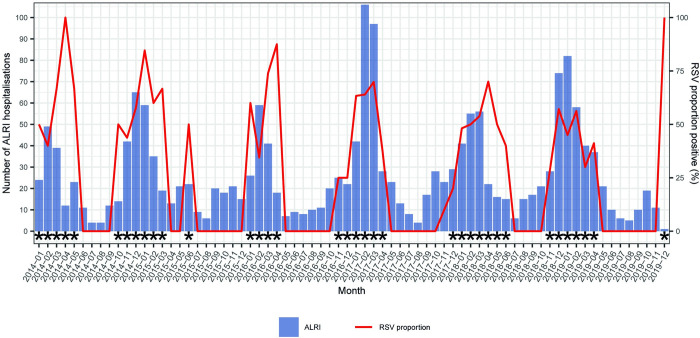
Seasonality of RSV during the study period. RSV = respiratory syncytial virus; ALRI = acute lower respiratory infection. Asterisks denote months with high RSV activity (>10% positivity rate).

The duration of RSV season (defined by >10% of RSV positivity rate) ranged from 4 to 7 months over the study years; RSV season could start as early as October and end as late as June (**Fig 2**). The onset of RSV season ranged from October to January (of the next year). Despite these year-on-year variations, January, February and March were all as part of the RSV seasons across the years and these three months also had the highest AAP compared with the rest months, jointly accounting for 75% of all RSV positives of the year (**S4 Table**).

### Stratified analysis by birth month

The stratified analysis of the hospitalisation rates showed that the risks for RSV-ALRI hospitalisations in the first year of life differed by birth month—infants who were born in November, December and January had the highest hospitalisation rates, ranging between 34.03 and 49.87 per thousand; by comparison, infants who were born in April, May and June had the lowest hospitalisation rates, ranging between 6.04 and 11.89 per thousand per year (**Table 2**).

**Table 2:** Annual hospitalisation rate (per thousand live births) in the first year of life by birth month in 2014–19.

Birth month	All-cause ALRI	RSV-associated ALRI
**January**	51.04 (42.06–61.36)	34.03 (25.84–43.99)
**February**	38.36 (30.07–48.23)	23.32 (15.84–33.10)
**March**	28.82 (21.94–37.18)	13.18 (7.53–21.40)
**April**	23.78 (17.14–32.15)	11.89 (5.44–22.57)
**May**	22.14 (15.81–30.14)	6.04 (2.22–13.14)
**June**	20.54 (14.80–27.76)	10.27 (4.70–19.49)
**July**	34.61 (27.27–43.32)	16.90 (10.46–25.84)
**August**	33.53 (26.51–41.85)	14.90 (9.10–23.02)
**September**	42.95 (35.02–52.14)	19.59 (12.80–28.70)
**October**	58.24 (48.62–69.20)	28.75 (20.44–39.30)
**November**	61.95 (51.87–73.41)	40.01 (30.67–51.29)
**December**	73.15 (62.19–85.48)	49.87 (39.23–62.52)

RSV = respiratory syncytial virus; ALRI = acute lower respiratory infection.

The distribution of RSV-ALRI hospitalisation rate by birth month was highly correlated with RSV seasonality. Correlation analyses showed that infants who were born 2–3 months before the RSV peak months had the highest risks for being hospitalised due to RSV-ALRI and that infants who were born 9 months before (or 3 months after) the RSV peak months had the lowest risks (**S1 Fig**).

**Fig 3:**
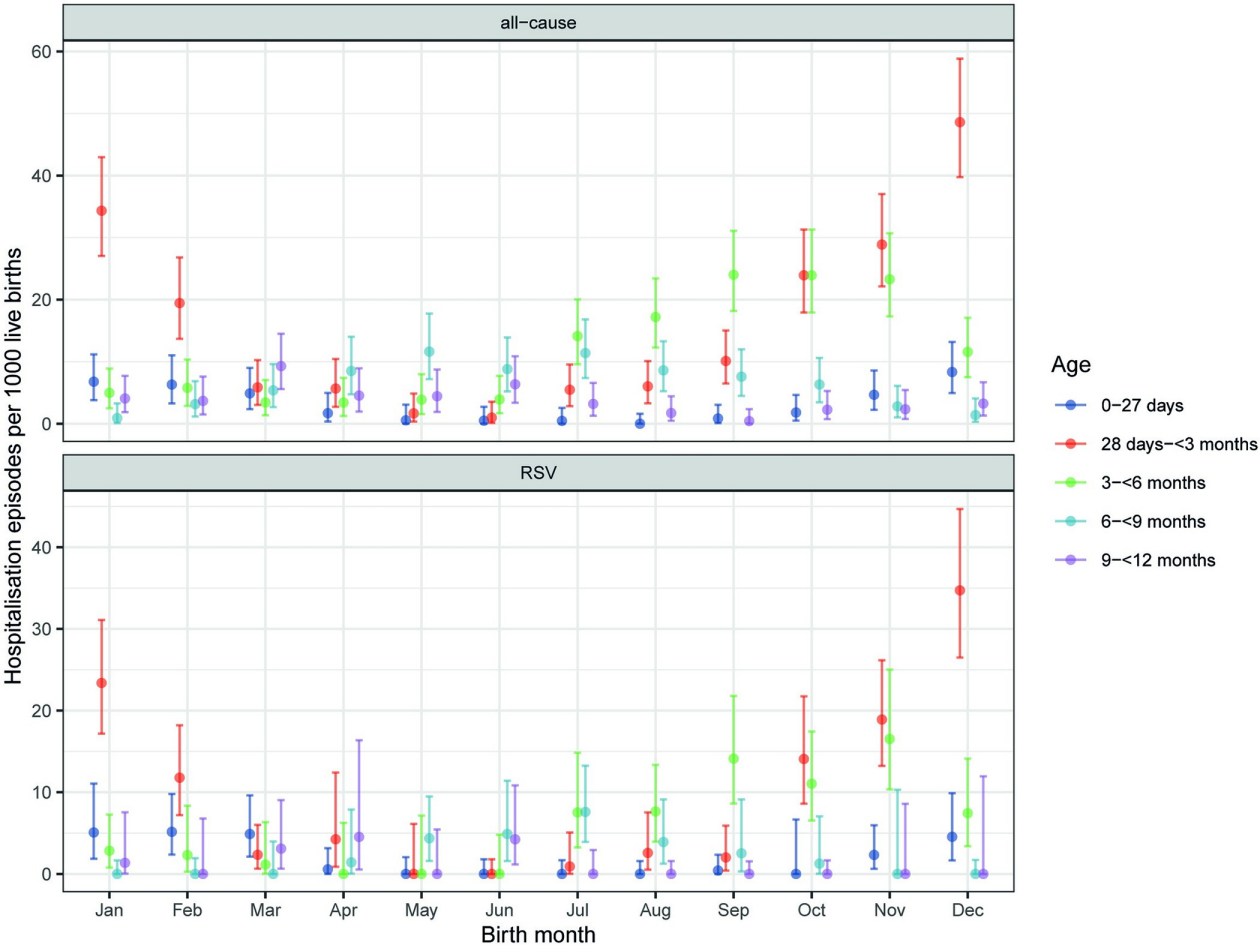
Annual hospitalisation rates of all-cause and RSV-associated ALRI in the first year of life (per 1000 live births) by birth month. RSV = respiratory syncytial virus; ALRI = acute lower respiratory infection.

Depending on the birth month of infants, the risk of RSV-ALRI hospitalisation peaked at different months of age. Infants who were born in December and January had the highest risk of RSV-ALRI hospitalisation at the age of 28 days–<3 months whereas infants who were born in September had the highest risk at the age of 3–<6 months (**Fig 3**).

## Discussion

In the present study, we estimated all-cause and RSV-ALRI hospitalisation rates in young children of different age bands in the Split-Dalmatia County, Croatia during 2014–19. The overall annualised hospitalisation rate in children under five years across the study years was 14.66 per thousand (95% CI: 14.01–15.34) for all-cause ALRI and was 7.56 per thousand (95% CI: 6.83–8.34) for RSV-ALRI. RSV-ALRI hospitalisation rate was higher in infants (<12 months) than children aged 12–<60 months, and was highest in infants aged 28 days–<3 months (61.15 per thousand per year, 95% CI: 52.91–70.31). Infants who were born in November, December and January (two to three months before RSV peak) had the highest hospitalisation rates during the first year of life.

Furthermore, to the best of our knowledge, our study is the first to highlight that RSV hospitalisation rate profile over chronological age differed by birth month. For example, while we found RSV-ALRI hospitalisation rate peaked at the age of 28 days–<3 months overall, infants born in September had the highest hospitalisation rate at the age of 3–<6 months; infants born in October and November also had considerably high RSV-ALRI hospitalisation rates at the age of 3–<6 months. This has several important implications for RSV immunisation strategies for infants. For RSV maternal vaccine, this poses a real challenge since the protection effect is very likely to have waned three months after birth. For RSV long-acting monoclonal antibody, the implication depends on the duration of protection—a birth-dose programme would be preferable if the duration of protection is long enough (e.g. ≥6 months) whereas a seasonal catch-up programme to vaccinate all infants, e.g. under three months of age, would be preferable if the duration of protection is not long enough (e.g. <6 months).

For the year 2019, we estimated that there were 1100 (95% CI: 840–1420) RSV-ALRI hospitalisations among children under five years in Croatia. This was broadly similar to the estimate from a global-disease-burden study using a modelling approach, although that estimate had a wider confidence interval (820, 95% CI: 220–1940) [18]. Similar to earlier studies [19–22] in Croatia, we documented some variations in RSV season in the study setting; the onset of RSV season ranged from October to January (the next year). Such year-on-year variation in RSV season needs to be fully considered when planning a seasonal catch-up RSV immunisation programme.

The strengths of our study include a clearly-defined catchment population that allows for the calculation of hospitalisation rate as well as the important exploration regarding the role of birth month in determining hospitalisation rate of RSV-ALRI among infants of finer age groups. However, we do acknowledge several limitations in our study. First, we used routine clinical database that was not primarily used for research. RSV testing was ordered at clinicians’ discretion and, as a result, was not available to all ALRI cases, introducing possible selection bias. This could bias upwards or downwards the RSV-ALRI hospitalisation rate estimates. Nonetheless, over 60% of ALRI cases in infants were tested for RSV and the proportion of testing was overall comparable across different birth months, which lends support to our estimate for the infant group and our main findings on RSV-ALRI hospitalisation by birth month. RSV testing was at a lower level in the year 2015 due to financial/technical reasons in our hospital, resulting in a wider uncertainty range of the estimate for that year. Moreover, only rapid antigen tests were used during the study period and these tests were likely to be less sensitive; as a result, this could underestimate the RSV-specific rates in our analysis. Second, we did not have enough RSV cases to explore whether birth month also plays an important role in RSV-ALRI hospitalisation rate in the second year of life. One of the hypotheses is that infants who were born in the “high-risk” months might also have a higher risk for RSV-ALRI hospitalisation in their second year of life. Third, we did not have enough severe ALRI cases for the aforementioned birth month analysis; we were also unable to further stratify our birth month analysis by season due to the scarcity of data. Fourth, the extrapolation of the hospitalisation rate in our study setting to the whole country of Croatia might be biased; our study setting had a higher under-five children mortality rate than the nationwide average (1.07/1000 *vs* 0.88/1000 in 2019) [14] and it is possible that the extrapolated estimate was biased upwards.

Despite these limitations, our study highlights the importance of birth month in planning RSV immunisation strategies for infants. Our estimates of RSV-ALRI hospitalisation rates in finer age bands also provide useful baseline data for future vaccination effectiveness analysis for various novel RSV prophylactic products.

## Supporting information

S1 Table Proportion of RSV testing by year, by severity and by age group. RSV = respiratory syncytial virus; ALRI = acute lower respiratory infection.
(DOCX)

S2 Table Proportion of RSV testing among infants by birth month. RSV = respiratory syncytial virus; ALRI = acute lower respiratory infection.
(DOCX)

S3 Table RSV proportion positive among ALRI tested for RSV by year, by severity and by age group. RSV = respiratory syncytial virus; ALRI = acute lower respiratory infection.
(DOCX)

S4 Table Monthly distribution of RSV-ALRI hospitalisations in 2014–19. ** Indicates peak RSV activity (the top three months with the highest accumulative RSV-ALRI hospitalisations); * indicates high RSV activity (with RSV positivity rate of >10%). RSV = respiratory syncytial virus; ALRI = acute lower respiratory infection.
(DOCX)

S1 Fig Results of correlation analyses showing the time lag between by birth month and by calendar month. The lag (in months) indicates number of months by which birth month is before calendar month. For example, a lag of one month means that the time series of birth month is one month before the time series of calendar month.
(TIF)
