# Supplementary material for: The role of birth month in the burden of hospitalisations for acute lower respiratory infections due to respiratory syncytial virus in young children in Croatia
Source: PLoS One. 2022 Sep 2;17(9):e0273962. doi: 10.1371/journal.pone.0273962 (PMC9439187; doi:10.1371/journal.pone.0273962)
Supplement: S1 Table — RSV = respiratory syncytial virus; ALRI = acute lower respiratory infection. (DOCX) [file pone.0273962.s001.docx]

# Table S1. Proportion of RSV testing by year, by severity and by age group

| Grouping | Total ALRI | ALRI tested for RSV | Proportion (%) |
| --- | --- | --- | --- |
| **By year** |  |  |  |
| 2014 | 299 | 97 | 32.4 |
| 2015 | 258 | 31 | 12.0 |
| 2016 | 256 | 86 | 33.6 |
| 2017 | 418 | 233 | 55.7 |
| 2018 | 366 | 178 | 48.6 |
| 2019 | 300 | 143 | 47.7 |
| **By severity** |  |  |  |
| All ALRI | 1897 | 768 | 40.5 |
| Severe ALRI | 131 | 54 | 41.2 |
| Very severe ALRI | 49 | 21 | 42.9 |
| **By age group** |  |  |  |
| <28d | 77 | 52 | 67.5 |
| 28d–<3m | 410 | 318 | 77.6 |
| 3–<6m | 306 | 182 | 59.5 |
| 6–<9m | 159 | 77 | 48.4 |
| 9–<12m | 93 | 31 | 33.3 |
| 0–<12m | 1045 | 660 | 63.2 |
| 12–<60m | 852 | 108 | 12.7 |

RSV = respiratory syncytial virus; ALRI = acute lower respiratory infection.
