# Supplementary material for: The role of birth month in the burden of hospitalisations for acute lower respiratory infections due to respiratory syncytial virus in young children in Croatia
Source: PLoS One. 2022 Sep 2;17(9):e0273962. doi: 10.1371/journal.pone.0273962 (PMC9439187; doi:10.1371/journal.pone.0273962)
Supplement: S2 Table — RSV = respiratory syncytial virus; ALRI = acute lower respiratory infection. (DOCX) [file pone.0273962.s002.docx]

# Table S2. Proportion of RSV testing among infants by birth month

| Birth month | Total ALRI | ALRI tested for RSV | Proportion (%) |
| --- | --- | --- | --- |
| January | 113 | 87 | 77.0 |
| February | 73 | 51 | 69.9 |
| March | 59 | 35 | 59.3 |
| April | 42 | 18 | 42.9 |
| May | 40 | 22 | 55.0 |
| June | 42 | 18 | 42.9 |
| July | 76 | 43 | 56.6 |
| August | 78 | 45 | 57.7 |
| September | 102 | 57 | 55.9 |
| October | 129 | 79 | 61.2 |
| November | 133 | 96 | 72.2 |
| December | 158 | 110 | 69.6 |

RSV = respiratory syncytial virus; ALRI = acute lower respiratory infection.
