# Supplementary material for: The role of birth month in the burden of hospitalisations for acute lower respiratory infections due to respiratory syncytial virus in young children in Croatia
Source: PLoS One. 2022 Sep 2;17(9):e0273962. doi: 10.1371/journal.pone.0273962 (PMC9439187; doi:10.1371/journal.pone.0273962)
Supplement: S3 Table — RSV = respiratory syncytial virus; ALRI = acute lower respiratory infection. (DOCX) [file pone.0273962.s003.docx]

# Table S3. RSV proportion positive among ALRI tested for RSV by year, by severity and by age group

| Grouping | ALRI tested for RSV | ALRI positive for RSV | Proportion (%) |
| --- | --- | --- | --- |
| **By year** |  |  |  |
| 2014 | 97 | 53 | 54.6 |
| 2015 | 31 | 19 | 61.3 |
| 2016 | 86 | 42 | 48.8 |
| 2017 | 233 | 134 | 57.5 |
| 2018 | 178 | 89 | 50.0 |
| 2019 | 143 | 59 | 41.3 |
| **By severity** |  |  |  |
| All ALRI | 768 | 396 | 51.6 |
| Severe ALRI | 54 | 21 | 38.9 |
| Very severe ALRI | 21 | 10 | 47.6 |
| **By age group** |  |  |  |
| <28d | 52 | 35 | 67.3 |
| 28d–<3m | 318 | 197 | 61.9 |
| 3–<6m | 182 | 96 | 52.7 |
| 6–<9m | 77 | 32 | 41.6 |
| 9–<12m | 31 | 10 | 32.3 |
| 0–<12m | 660 | 370 | 56.1 |
| 12–<60m | 108 | 26 | 24.1 |

RSV = respiratory syncytial virus; ALRI = acute lower respiratory infection.
