# Supplementary material for: The role of birth month in the burden of hospitalisations for acute lower respiratory infections due to respiratory syncytial virus in young children in Croatia
Source: PLoS One. 2022 Sep 2;17(9):e0273962. doi: 10.1371/journal.pone.0273962 (PMC9439187; doi:10.1371/journal.pone.0273962)
Supplement: S4 Table — ** Indicates peak RSV activity (the top three months with the highest accumulative RSV-ALRI hospitalisations); * indicates high RSV activity (with RSV positivity rate of >10%). RSV = respiratory syncytial virus; ALRI = acute lower respiratory infection. (DOCX) [file pone.0273962.s004.docx]

# Table S4. Monthly distribution of RSV-ALRI hospitalisations in 2014–19

| Month | Annual average percentage of RSV-ALRI hospitalisations  (denominator = total number of RSV positive counts during the study period;  numerator = number of RSV positive counts in each month) | RSV proportion positive among ALRI hospitalisations  (denominator = number of ALRI hospitalisations receiving RSV tests in each month;  numerator = number of RSV positive counts in each month) |
| --- | --- | --- |
| January** | 19.9 (79/396) | 54.1 (79/146) |
| February** | 27.3 (108/396) | 54.3 (108/199) |
| March** | 27.5 (109/396) | 61.9 (109/176) |
| April* | 8.1 (32/396) | 56.1 (32/57) |
| May* | 2.5 (10/396) | 37.0 (10/27) |
| June* | 0.8 (3/396) | 33.3 (3/9) |
| July | 0 (0/396) | 0 (0/2) |
| August | 0 (0/396) | 0 (0/5) |
| September | 0 (0/396) | 0 (0/8) |
| October | 0.3 (1/396) | 7.7 (1/13) |
| November* | 2.8 (11/396) | 25.6 (11/43) |
| December* | 10.9 (43/396) | 51.8 (43/83) |
| Total | 100 (396/396) | 51.6 (396/768) |

** Indicates peak RSV activity (the top three months with the highest accumulative RSV-ALRI hospitalisations); * indicates high RSV activity (with RSV proportion positive of >10%). RSV = respiratory syncytial virus; ALRI = acute lower respiratory infection.
