# Supplementary figures and images for: The role of birth month in the burden of hospitalisations for acute lower respiratory infections due to respiratory syncytial virus in young children in Croatia
Source: PLoS One. 2022 Sep 2;17(9):e0273962. doi: 10.1371/journal.pone.0273962 (PMC9439187; doi:10.1371/journal.pone.0273962)

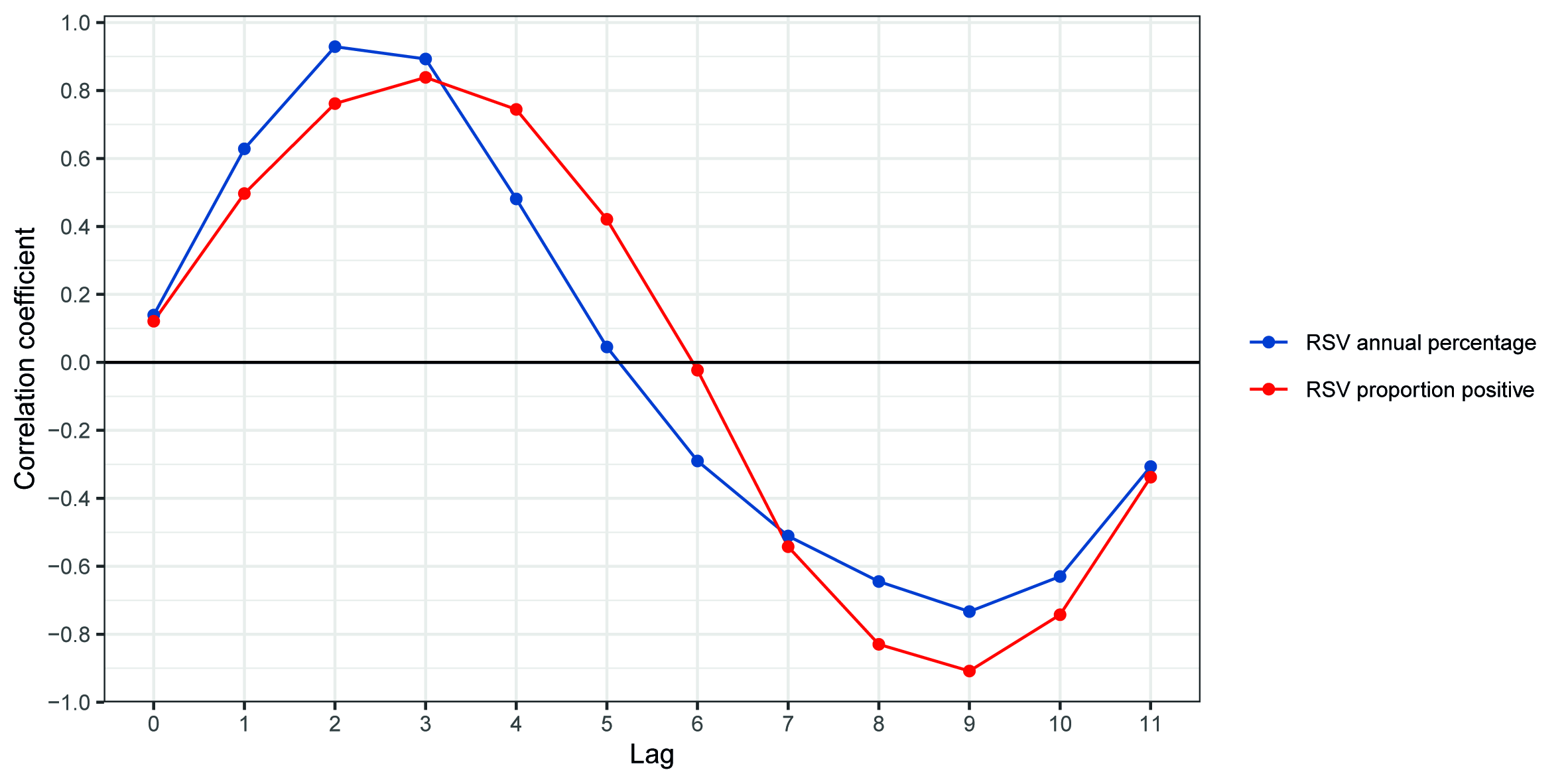

Supplement: S1 Fig — The lag (in months) indicates number of months by which birth month is before calendar month. For example, a lag of one month means that the time series of birth month is one month before the time series of calendar month. (TIF) [file pone.0273962.s005.tif]
